# Efficacy of Early Endoscopic Ultrasound-Guided Transluminal Drainage for Postoperative Pancreatic Fistula

**DOI:** 10.1155/2021/6691705

**Published:** 2021-01-25

**Authors:** Nao Fujimori, Takashi Osoegawa, Akira Aso, Soichi Itaba, Yosuke Minoda, Masatoshi Murakami, Kazuhide Matsumoto, Katsuhito Teramatsu, Yu Takamatsu, Takehiro Takaoka, Takamasa Oono, Eikichi Ihara, Tomoharu Yoshizumi, Takao Ohtsuka, Masafumi Nakamura, Yoshihiro Ogawa

**Affiliations:** ^1^Department of Medicine and Bioregulatory Science, Graduate School of Medical Sciences, Kyushu University, Fukuoka, Japan; ^2^Department of Gastroenterology and Metabolism, Graduate School of Medical Sciences, Kyushu University, Fukuoka, Japan; ^3^Department of Surgery and Science, Graduate School of Medical Sciences, Kyushu University, Fukuoka, Japan; ^4^Department of Surgery and Oncology, Graduate School of Medical Sciences, Kyushu University, Fukuoka, Japan

## Abstract

**Background:**

Endoscopic ultrasound-guided transluminal drainage (EUS-TD) is generally performed 4 weeks after disease onset for evacuating pancreatic fluid collections. However, the optimal timing for conducting the procedure in those diagnosed with postoperative pancreatic fistula (POPF) has not been established. We aimed to elucidate the efficacy and safety of early EUS-TD procedures for treating POPF.

**Methods:**

We retrospectively reviewed patients diagnosed with POPF who underwent EUS-TD in the Kyushu University Hospital between 2008 and 2019. Clinical features were comparatively analyzed between the two patient groups who underwent either early (≤15 days postoperatively) or late (>15 days postoperatively) EUS-TD. Factors prolonging hospital stay were also analyzed using Cox proportional hazard models.

**Results:**

Thirty patients (median age, 64.5 years) were enrolled. The most common initial operation was distal pancreatectomy with splenectomy (60.0%). Median size of POPF was 69.5 (range, 38–145) mm, and median time interval between surgery and EUS-TD was 17.5 (range, 3–232) days. Totally, 47% patients underwent early EUS-TD. Rates of technical success, clinical success, and complications were 100%, 97%, and 6.9%, respectively. No recurrence of POPF occurred during a median follow-up period of 14 months. Clinical characteristics and outcomes were comparable between the early and late drainage patient groups, except for the rates of infection and nonencapsulation of POPF, which were significantly higher in the early drainage group. Performing simultaneous internal and external drainage (hazard ratio (HR): 0.31; 95% confidence interval (CI): 0.11–0.93, *p*=0.04) and conducting ≥2 treatment sessions (HR: 0.26; 95% CI: 0.08–0.84, *p*=0.02) were significantly associated with prolonged hospitalization after EUS-TD.

**Conclusions:**

EUS-TD is a safe and effective method for managing POPF, regardless of when it is performed in the postoperative period. Once infected POPF occurs, clinicians should not hesitate to perform EUS-TD even within 15 days of the initial operation.

## 1. Introduction

Postoperative pancreatic fistula (POPF), one of the most serious postoperative complications occurring after abdominal surgery, should be treated appropriately via percutaneous or endoscopic drainage [1–3]. POPF often causes abdominal pain, infection, and internal hemorrhage, making timely management extremely essential for improving clinical outcomes in affected patients [2, 4]. Endoscopic ultrasound-guided transluminal drainage (EUS-TD) of POPF had been initially recognized as an alternative treatment modality for cases in which percutaneous drainage was ineffective or technically difficult to perform. However, it is now recommended as the first-line treatment for managing postoperative fluid collections, including POPF, considering many advantages of internal over percutaneous drainage [5–13].

Pancreatic fluid collections (PFCs) may present as pancreatic pseudocysts (PPC) or as walled-off necrosis (WON) 4 weeks after onset of acute pancreatitis. EUS-TD has already been established as the best method for draining PFCs arising secondary to acute pancreatitis [14, 15] and is strongly recommended for draining both infected PPCs and WON lesions [16, 17]. EUS-TD is usually performed 4 weeks after the onset of acute pancreatitis to allow for encapsulation of these lesions, so that margins of a PFC can be clearly defined on imaging [16]. However, it is unknown whether the same concept of delayed treatment is applicable to management of POPF.

In addition to the lack of clarity regarding the optimal timing for performing the procedure, it is also unclear whether a double-pigtailed plastic stent (PS) or a metallic stent (such as lumen-apposing metal stents (LAMS)) should be employed during EUS-TD of POPF. Recently, 2 studies on EUS-TD using LAMS for treatment of postoperative fluid collections have been reported [18, 19]. Yang et al. achieved high rates of technical and clinical success of EUS-TD procedures performed using LAMS at 96.8% and 91.9%, respectively; however, only 16% of the study patients received the treatment during the initial 30-day postoperative period [18]. The optimal timing and device for performing EUS-TD for POPF have not been fully elucidated, and only few reports have especially focused on outcomes of early, postoperative drainage procedures [9, 11, 20].

In this study, we aimed to investigate the efficacy and safety of EUS-TD for evacuating POPF and also to evaluate the clinical utility of such drainage procedures during the early postoperative period.

## 2. Materials and Methods

### 2.1. Study Population and Data Collection

We retrospectively recruited all patients diagnosed with POPF who underwent EUS-TD at the Kyushu University Hospital between May 2008 and August 2019. Diagnosis of POPF was confirmed in all patients using computed tomography (CT) scans. The timing of drainage was determined by consensus among the endoscopists and surgeons, according to the size and location of POPF and the patients' symptoms. We collated clinical data of all patients including age, sex, presenting symptoms, initial surgical procedure, primary postoperative histopathological diagnosis, imaging findings of POPF, indication and details of EUS-TD, levels of technical and clinical success achieved, and complications. We obtained informed consent for conducting EUS-TD from all patients. The study protocol was approved by the Ethics Committee of the Kyushu University Hospital (approval no. 2019-595).

### 2.2. EUS-TD Procedure

**Figure 1:**
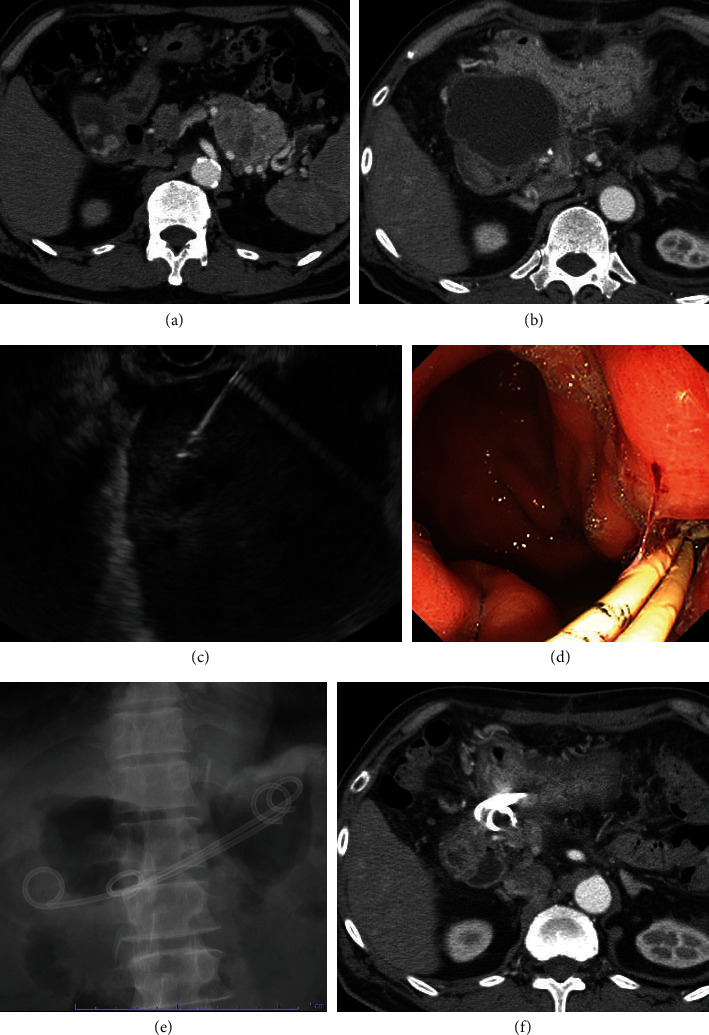
Clinical images of a representative patient who underwent endoscopic ultrasound-guided transluminal drainage (EUS-TD) for postoperative pancreatic fistula (POPF). (a) Preoperative computed tomography (CT) scan reveals a tumor in the pancreatic tail, diagnosed as a pancreatic neuroendocrine tumor. The patient underwent distal pancreatectomy. (b) CT scan performed 14 days after surgery shows an infected POPF of 75 mm diameter. (c) EUS imaging performed on day 15 after surgery shows peripancreatic fluid collection, into which a 19G needle is inserted via a transgastric route. (d) and (e) Two double-pigtailed plastic stents were inserted and can be observed on endoscopic imaging (d) and under fluoroscopic (e) guidance. (f) CT scan performed 2 weeks after EUS-TD reveals resolution of the POPF.

EUS-TD was performed with the patient under conscious sedation with midazolam and pentazocine hydrochloride. Prophylactic antibiotics were intravenously administered to all patients, either before or during the EUS-TD procedure. A convex array echoendoscope (GF-UCT240-AL5 or GF-UCT260-AL5; Olympus Medical Systems, Tokyo, Japan), equipped with an ultrasound processor (SSD-*α*5, ALOKA Co., Ltd, Tokyo, Japan, or Pro-Sound F75, Hitachi Aloka Medical, Tokyo, Japan) was used, as described previously [21]. After insertion of a 19G needle into the POPF under EUS guidance, the fluid within was partially aspirated for bacteriological culture analysis and evaluation of its amylase level. Whenever PS placement was intended, a 0.025-inch guidewire was inserted and looped into the cavity. Thereafter, the fistula tract was dilated using either a noncauterizing device (Hurricane RX or CRE, Boston Scientific, Natick, MA; Ren, Kaneka, Tokyo, Japan; ES dilator, Zeon Medical Inc., Tokyo, Japan) or a cautery (Cysto-Gastro-Set; Endo-Flex GmbH, Voerde, Germany). This was followed by insertion of a 7-Fr size, double-pigtailed PS and/or a 5-6-Fr nasocystic drainage tube along the guidewire, into the lumen. In certain patients, only aspiration was performed until the entire liquid component was evacuated under EUS guidance, as indicated. In patients requiring LAMS (Hot AXIOS, Boston Scientific) placement, the metal stent was inserted into the POPF cavity, followed by deployment of its distal flange under EUS guidance and subsequently of the proximal flange under endoscopic guidance [18]. The type of drainage procedure was chosen at the discretion of the endoscopist, and the route of drain insertion (transgastric or transduodenal) was selected according to the anatomical location of the POPF. Simultaneous internal and external drainage (placement of both a PS and nasocystic tube) was performed according to the discretion of the endoscopist if purulent and/or viscous fluid was aspirated. If clinical improvement was not observed after primary EUS-TD (e.g., persistent abdominal pain, fever, and elevated inflammatory response), additional endoscopic procedures such as dilation of the fistula using a balloon-catheter and stent-exchange or insertion of additional PS were performed. Clinical images of a representative patient who underwent EUS-TD for evacuation of POPF are shown in Figure 1.

### 2.3. Definitions

An encapsulated POPF was defined as fluid collection with a well-defined inflammatory wall on CT [11, 16]. Technical success was defined as the successful completion of the intended procedure, i.e., achievement of either complete aspiration of the fluid component or the successful endoscopic placement of a double-pigtailed PS/LAMS in the proper position. Clinical success was defined as the shrinkage of POPF on the follow-up CT scan along with resolution of the patients' associated symptoms, such as fever and abdominal pain, by endoscopic treatments only. Patients requiring further percutaneous drainage under US or CT guidance were defined as clinical failures. Early and late drainage for POPF was defined as EUS-TD performed within and after 15 days of the initial pancreatic procedure, respectively.

### 2.4. Statistical Analysis

Statistical analyses were performed using the JMP ver.14 (SAS Institute Inc.) software. Differences in clinical characteristics between patients who underwent early drainage of POPF and those who received the treatment later were evaluated using the chi-squared test, Fisher's exact test, or the unpaired *t*-test, as appropriate. Univariate and multivariate analyses were conducted using Cox proportional hazard models to identify factors predictive of prolonged hospitalization. The level of significance was set at *p* < 0.05.

## 3. Results

### 3.1. Patient Characteristics

**Figure 2:**
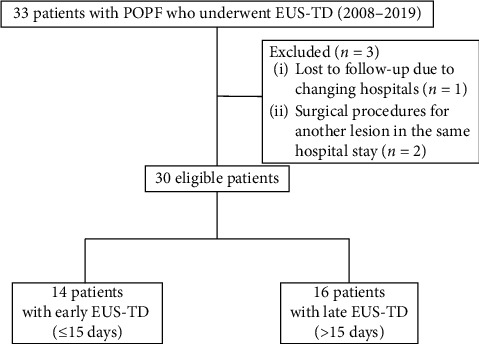
Flowchart of enrolled study patients. POPF, postoperative pancreatic fistula; EUS-TD, endoscopic ultrasound-guided transluminal drainage.

Figure 2 is a flowchart summarizing enrollment of all study patients. Totally, 33 patients with POPF who underwent EUS-TD in the Kyushu University Hospital between 2008 and 2019 were enrolled, and their data were reviewed retrospectively. Three patients were excluded, as they changed hospitals (lost to follow-up) or underwent surgical procedures for another lesion during the same hospital stay. Finally, data of 30 patients were analyzed, and their clinical characteristics are summarized in Table 1. The most common primary surgical procedure was distal pancreatectomy with splenectomy (60.0%). The median size of POPF was 69.5 (range 38–145) mm, and median time interval between surgery and EUS-TD was 17.5 (range 3–232) days. Mostly, POPF developed in the pancreatic body or tail and therefore was drained via the transgastric route. Nine (30%) patients required ≥2 treatment sessions. Early drainage (during the ≤15-day postoperative period) was performed in 47% patients. Rates of technical success, clinical success, and occurrence of complications were estimated at 100%, 97%, and 6.9%, respectively. Both patients who experienced postprocedural complications had bleeding after EUS-TD and required angiography to facilitate hemostasis. A cautery device was used to dilate the fistula tract followed by PS placement during the EUS-TD procedure in both cases. No recurrence of fluid collections was observed during a median follow-up period of 14 months.

### 3.2. Comparison of Clinical Characteristics between Early and Late Drainage Groups

We compared clinical characteristics between patients who underwent early EUS-TD (early drainage group) for POPF and those who received the treatment later (late drainage group) to evaluate the safety and efficacy of the procedure when performed within 15 days postoperatively (Table 2). A comparative analysis of clinical characteristics between both groups of patients showed no significant differences, except for the presence of infection and encapsulation of POPF. Other factors, including the primary surgical procedure, features of POPF, and the type of EUS-TD procedure used, were comparable between both groups. However, the incidence of infection of POPF in patients who underwent early drainage was significantly higher than that observed in those in the late drainage group (14/14 vs. 5/16, *p* < 0.0001). The rate of encapsulation of POPF in the early EUS-TD group was significantly lower than that in the late EUS-TD group (8/14 vs. 15/16, *p*=0.02). Median period of hospitalization after EUS-TD in the early and late drainage groups was 23.5 and 21.4 days, respectively (*p*=0.75). Clinical outcomes including rates of technical success, clinical success, and complications were also equivalent in both groups.

### 3.3. Predictive Factors for Prolonged Hospital Stay after EUS-TD

Finally, we investigated factors predictive of prolonged hospital stay after EUS-TD using Cox proportional hazards models. Based on the results of the univariate analyses, multivariate analysis was performed for three significant variables (*p* < 0.01), such as simultaneous internal and external drainage, drainage without LAMS, and requirement of ≥2 treatment sessions. The multivariate analyses showed that conducting internal and external drainage simultaneously (hazard ratio (HR): 0.31; 95% confidence interval (CI): 0.11–0.93, *p*=0.04) and performing ≥2 treatment sessions (HR 0.26; 95% CI: 0.08–0.84, *p*=0.02) were factors significantly associated with prolonged hospitalization after EUS-TD (Table 3). Timing of EUS-TD (performed either earlier or later in the postoperative period) and encapsulation of the POPF did not influence the length of postprocedural hospitalization.

## 4. Discussion

In this study, we demonstrated that EUS-TD was an effective and safe treatment for POPF, even during the early postoperative period (≤15 days postoperatively). This is clinically important because unlike a delay of 4 weeks from onset of acute pancreatitis, as advised for EUS-TD performed in patients with other PFCs such as PPC and WON [16, 17], the optimal postoperative time interval for conducting the procedure in those diagnosed with POPF has not yet been determined.

Endoscopists tend to hesitate to perform EUS-TD during the early postoperative period, considering immaturity of adhesions between POPF and adjoining gastric or duodenal walls. Furthermore, POPF is not properly encapsulated within this time interval. Several previous studies on EUS-TD for POPF have reported a mean timing of drainage of >1 month postoperatively [7, 9, 10, 18]. At our hospital, the procedure was performed at a median time interval of 17.5 days postoperatively. Despite this period, being shorter than that reported previously, rates of technical and clinical success and those of complications that occurred in our cohort were within an acceptable range. These results indicate that EUS-TD is an effective method for treating POPF, regardless of when it is performed during the postoperative period, a feature that is also characteristic of conventional percutaneous drainage procedures.

Theoretically, early drainage of POPF using EUS-TD may be associated with an increased rate of complications and decreased therapeutic efficacy, considering lack of encapsulation of the POPF and immature adhesions connecting it to the gastric/duodenal walls. Therefore, we compared clinical characteristics and outcomes between patients who underwent early (performed in ≤15 postoperative days) and late (performed after >15 postoperative days) drainage of POPF. We found both groups to differ only with respect to occurrence of infection and encapsulation of the POPF. Previous studies have also confirmed that performing early EUS-TD neither increases complication rates nor reduces therapeutic efficacy [11, 13]. Recently, Storm et al. reported that the efficacy and safety of acute and early EUS-TD performed within 30 days postoperatively to relieve postsurgical fluid collections were comparable with those associated with a delayed procedure (performed after >30 days postoperatively) [20]. They achieved high technical and clinical success rates of 100% and 93%, respectively, with early EUS-TD. Furthermore, they also reported the efficacy and safety of acute EUS-TD (within 14 days postoperatively) for POPF in 20 patients. Although encapsulation of POPF was not mentioned, they achieved extremely high technical/clinical success rates with only 3 mild adverse events in patients undergoing acute EUS-TD. In another study by Tamura et al. [11], EUS-TD involving external drainage using a nasocystic drainage was effective for drainage of noncapsulated POPF (within 28 days postoperatively). There were also no severe complications after EUS-TD for noncapsulated POPF. Early EUS-TD for POPF may be more effective because POPF may mainly contain a liquid component during this period. Furthermore, POPF may become adherent to the gastric/duodenal walls earlier than other PFCs due to postoperative inflammation and the presence of infection within the POPF itself. Considering these possible explanations and the results from our own and previous studies, we surmised that it is not always necessary to delay the procedure for 4 weeks until both maturity of adhesions and encapsulation of POPF have occurred. Nevertheless, further evaluation is essential to validate the efficacy and safety of early EUS-TD. We believe that endoscopists should not hesitate to perform EUS-TD, especially in patients with infected POPF, even during the early postoperative period (≤15 days). However, we also recommend that only expert endoscopists should perform this delicate procedure. Clear detection of POPF on EUS imaging may be challenging even for expert endoscopists because it may be technically difficult to distinguish POPF from postoperative inflammatory changes, especially in patients undergoing early postoperative drainage.

Evaluation of EUS-TD techniques showed that performing internal and external drainage simultaneously was related to prolonged postprocedural hospitalization. However, this combined approach allowed us to evaluate fluid output and is therefore especially useful in patients with infected PFCs. Tamura et al. [11] also reported on the utility of external drainage using a nasocystic tube for drainage of POPF. Although endoscopists should take the possibility of a slightly longer hospital stay into consideration, implementing this technique is a treatment option for POPF.

While efficacy of LAMS for drainage of PFCs is nearly established, its application for draining POPF is still controversial. In our study patients, we mainly used double-pigtailed PS for EUS-TD because insertion of LAMS was not covered by the Japanese insurance system. LAMS was utilized only in 4 (13.3%) patients in our study, all of whom had large-sized (median, 83 mm) POPF with relatively clear margins. Recently, two studies on the use of LAMS for draining POPF demonstrated its efficacy in patients with large and encapsulated lesions (clinical success rate, ∼90%) [18, 19]. Conversely, a recent randomized trial by Bang et al. [22, 23] did not find insertion of LAMS to be superior to that of PS for drainage of WON lesions. They reported that an increase in occurrence of stent-related adverse events occurred ≥3 weeks after placement of LAMS. Our study, which mainly utilized PS, revealed good efficacy and safety of these stents. Furthermore, POPF is sometimes not deep enough to allow for safe insertion of LAMS into the cavity, especially in patients undergoing early EUS-TD. Donatelli et al. also reported that PS may be safer to use than LAMS, during EUS-TD procedures performed without conducting endoscopic necrosectomy because the former are soft, mobile, and pliable, thus reducing trauma to surrounding structures [10]. We found no reports involving direct comparisons of LAMS with PS in EUS-TD for POPF. Therefore, either PS or LAMS should be selected on a case-to-case basis.

This study has several limitations. First, we retrospectively analyzed only those patients with POPF who had been treated using EUS-TD. A comparative analysis of EUS-TD with percutaneous drainage was not performed in this study. Second, this was a single-center study and the sample size was small, factors that may impact the generalizability of our findings. Finally, various endoscopic techniques and devices were applied due to the relatively long study period, which may affect clinical outcomes and hospital stay after EUS-TD. While further prospective studies are required to validate true utility of early EUS-TD for managing POPF, our results have important implications for clinical decision-making in patients developing this complication following pancreatic surgery.

In conclusion, our single-center study elucidated that EUS-TD is a safe and effective method for managing POPF, regardless of when it is performed in the postoperative period. Once infected POPF occurs, clinicians should not hesitate to perform EUS-TD, even during the first 15 days after the initial operation.

## Figures and Tables

**Table 1:** Characteristics of enrolled patients.

Patient characteristics	Values
Age (median (range))	64.5 (10–87)

Sex, male/female, *n*	18/12

Indication for surgery	
PDAC	9
NET	6
SPN	5
IPMN	4
LDLT	2
LC	1
LEC	1
Liposarcoma	1
MPD stricture	1

Type of initial surgery	
DP with splenectomy	18
SPDP	5
Enucleation	2
LDLT	2
DP-CAR	1
Splenectomy	1
Retroperitoneal tumor resection	1
With/without splenectomy	22/8
Open/laparoscopic	13/17

Indication for POPF drainage	
Infection	19
Pain	7
Asymptomatic	2
Others	2
Size of POPF (mm, median (range))	69.5 (38–145)

Location of POPF	
Ph	4
Pbt	26

Drainage approach	
Transgastric	29
Transduodenal	1

Type of drainage	
Simultaneous internal and external drainage	12
Internal drainage only	15
Aspiration	3
Plastic stent/LAMS	23/4

Number of treatment sessions	
1	21
2	6
3	2
4	1

Period between surgery and EUS-TD (days, median (range))	17.5 (3–232)
Hospital stay period after EUS-TD (days, median (range))	16.5 (5–88)
Technical success (*n*, %)	30 (100)
Clinical success (*n*, %)	29 (97)
Complications (*n*, %)	2 (6.9)
Observation period (months, median (range))	14 (0.6–117)

POPF, postoperative pancreatic fistula; PDAC, pancreatic ductal adenocarcinoma; NET, neuroendocrine tumor; SPN, solid pseudopapillary neoplasm; IPMN, intraductal papillary mucinous neoplasm; LDLT, living donor liver transplantation; LC, liver cirrhosis; LEC, lymphoepithelial cyst; MPD, main pancreatic duct; DP, distal pancreatectomy; SPDP, spleen-preserving distal pancreatectomy; DP-CAR, distal pancreatectomy with celiac axis resection; LAMS, lumen-apposing metal stent; EUS-TD, EUS-guided transluminal drainage.

**Table 2:** Comparison of clinical characteristics between the early and late drainage patient groups.

Clinical characteristics	Early drainage (≤15 days)	Late drainage (>15 days)	*p* value
Number of patients	14	16	
Median days from surgery to EUS-TD	10.5 (3–15)	34.5 (17–232)	
Gender (male/female)	9/5	9/7	0.65
Age (years, mean)	63.0 ± 5.2	56.6 ± 4.8	0.38

Indication for surgery			0.87
PDAC	4	5	
Not PDAC	10	11	

Surgery			0.83
With splenectomy	10	12	
Without splenectomy	4	4	

Open/laparoscopic			0.96
Open	6	7	
Laparoscopic	8	9	

Indication for POPF drainage			<0.0001
With infection	14	5	
Without infection	0	11	
Size of POPF (mm, mean)	66.1 ± 7.3	78.8 ± 6.8	0.21

Location of POPF			0.89
Ph	2	2	
Pbt	12	14	

Encapsulation of POPF			0.02
Absent	6	1	
Present	8	15	

Drainage approach			0.28
Transgastric	13	16	
Transduodenal	1	0	

Type of drainage			0.77
Simultaneous internal and external drainage	6	6	
Without external drainage (aspiration only)	8 (2)	10 (1)	

Type of stent used			0.35
LAMS	1	3	
Not LAMS	13	13	

Number of procedures			0.87
1	10	11	
2 or more	4	5	

Hospital stay after EUS-TD (days, mean (range))	23.5 ± 4.9	21.4 ± 4.6	0.75
Technical success (*n*, %)	14 (100)	16 (100)	
Clinical success (*n*, %)	13 (93)	16 (100)	0.28
Complications (*n*, %)	1 (7.1)	1 (6.3)	0.92

EUS-TD, EUS-guided transluminal drainage; PDAC, pancreatic ductal adenocarcinoma; POPF, postoperative pancreatic fistula; LAMS, lumen-apposing metal stent.

**Table 3:** Univariate and multivariate analyses of factors influencing prolonged hospitalization after EUS-TD.

Factor	Univariate			Multivariate		
*n* = 30	HR	95% CI	*p* value	HR	95% CI	*p* value
Sex (male)	0.92	0.51–2.33	0.82			
Age > 65	1.28	0.60–2.73	0.52			
PDAC	0.87	0.39–1.94	0.74			
Splenectomy	0.81	0.35–1.87	0.62			
Laparoscopic surgery	1.44	0.67–3.02	1.44			
Infection of POPF	1.04	0.49–2.22	0.91			
Size of POPF >70 mm	0.96	0.46–2.00	0.92			
Location, Ph	1.90	0.64–5.63	0.25			
Encapsulation of POPF	0.89	0.35–2.22	0.80			
Simultaneous internal and external drainage	0.18	0.06–0.51	0.0012	0.31	0.11–0.93	0.04
Not using LAMS	0.17	0.05–0.58	0.005	0.33	0.10–1.13	0.08
Total number of procedures >1	0.17	0.06–0.51	0.0016	0.26	0.08–0.84	0.02
Early drainage (within 15 days after surgery)	0.90	0.43–1.88	0.77			

EUS-TD, EUS-guided transluminal drainage; HR, hazard ratio; CI, confidence interval; PDAC, pancreatic ductal adenocarcinoma; POPF, postoperative pancreatic fistula; LAMS, lumen-apposing metal stent.

## Data Availability

The research data used to support the findings of this study are available from the corresponding author upon request.
